# Circulating blood microbiome biomarkers as *liquid biopsy* in breast cancer

**DOI:** 10.1186/s13046-026-03782-8

**Published:** 2026-08-11

**Authors:** William Jacot, Laure Cayrefourcq, Catherine Dunyach-Remy, Marta Jarlier, Gerald Lossaint, Véronique D’Hondt, Théo Pastre, Françoise Garima, Blandine Massemin, Séverine Guiu, Anne Marie Gorce Dupuy, Jean-Paul Cristol, Jean-Philippe Lavigne, Catherine Alix-Panabières

**Affiliations:** 1 Department of Medical Oncology, Institut du Cancer de Montpellier (ICM), Montpellier University, Montpellier, France; 2 Institut de Recherche en Cancérologie de Montpellier (IRCM), INSERM U1194, Montpellier University, Montpellier, France; 3 Laboratory of Rare Human Circulating Cells, University Medical Center of Montpellier, SUB, 371, Avenue du Doyen Gaston Giraud, Cedex, 34295 Montpellier, France; 4 CREEC/CANECEV, MIVEGEC (CREES), Université de Montpellier, CNRS, IRD, Montpellier, France; 5 European Liquid Biopsy Society (ELBS), Hamburg, Germany; 6 Department of Microbiology and Hospital Hygiene, VBIC, INSERM, Univ Montpellier, CHU Nîmes, Nîmes, France; 7 Biometrics Unit, Institut du Cancer Montpellier (ICM), Montpellier University, Montpellier, France; 8 Clinical and Translational Research Department (DRCI), Montpellier Cancer Institute (ICM), University of Montpellier, Montpellier, France; 9 Biological Resources Center, Montpellier Cancer Institute (ICM), University of Montpellier, Montpellier, France; 10 Laboratory of Biochemistry, University Medical Center of Montpellier, Montpellier, France

**Keywords:** Breast cancer, Blood microbiome, LBP, sCD14, I-FAPB, CTCs, PD-L1, Liquid biopsy

## Abstract

**Background:**

Circulating tumor material can already be used to help in cancer management, but host–microbiota interactions may add complementary value. This study evaluated a “liquid microbiopsy”, combining cell-free microbial DNA (cf-mbDNA) with host biomarkers of microbial translocation/permeability (LBP, sCD14, I-FABP), to refine staging and prognosis in breast cancer.

**Methods:**

We analyzed 136 individuals: 58 metastatic breast cancer (MBC), 58 early breast cancer (EBC), and 20 healthy donors. Plasma cf-mbDNA was quantified by 16S rRNA qPCR with extraction/run negative controls; LBP, sCD14, I-FABP by ELISA; and CRP by immunoturbidimetry. Circulating tumor cells (CTCs) and PD-L1 on CTCs were assessed by CellSearch® and immunostaining. Diagnostic performance was assessed by area under the ROC curve. In MBC, progression-free survival (PFS) and overall survival (OS) were analyzed using Kaplan–Meier and Cox models.

**Results:**

LBP discriminated EBC from MBC with an AUC 0.92, with a slight improvement in the 3- or 5-marker models (AUC 0.936–0.939). For EBC *vs* healthy donors, CRP showed modest performance (AUC 0.73), and marker combinations offered no improvement.. In MBC (median follow-up 77.5 months), median PFS and OS were 4.5 and 14.5 months, respectively. In univariate analysis, shorter PFS was associated with tumor subtype, > 3 prior treatments, > 2 metastatic sites, CTCs ≥ 5, and PD-L1–positive CTCs detection. In multivariate analysis, sCD14 and I-FABP independently predicted PFS, while sCD14 was independently associated with OS.

**Conclusions:**

A measurable host–microbiota–barrier axis (LBP, sCD14, I-FABP) provided stage-related and prognostic information complementary to CTC/PD-L1 status, whereas cf-mbDNA load alone showed limited discriminative value. These findings support liquid microbiopsy as a complementary extension of liquid biopsy but must be considered as exploratory, and warrant multicenter validation in larger independent cohorts with standardized pre-analytics and low-biomass controls.

**Trial registration:**

NCT03449264; NCT02866149.

**Supplementary Information:**

The online version contains supplementary material available at https://doi.org/10.1186/s13046-026-03782-8.

## Background

Metastatic dissemination, the process whereby tumor cells spread from the primary lesion to distant organs through the bloodstream, remains the leading cause of cancer-related mortality. Over the past two decades, the detection and molecular characterization of circulating tumor cells (CTCs) have substantially advanced our understanding of hematogenous metastatic progression [1]. The term ‘*liquid biopsy’*, first introduced in 2010 to describe the analysis of CTCs in peripheral blood [2], has since expanded to encompass a broader range of circulating biomarkers. These include *(i)* tumor-derived components such as circulating cell-free tumor DNA (ctDNA), circulating cell-free RNA (mRNA and noncoding RNA), extracellular vesicles (oncosomes), tumor-educated platelets, and tumor-associated proteins; and *(ii)* tumor-induced circulating biomarkers, including immune cells and soluble immune mediators such as cytokines and interleukins [3]. The liquid biopsy paradigm has also been extended beyond blood to other biological fluids, including saliva, urine, bone marrow, and cerebrospinal fluid [4]. Clinically, liquid biopsies offer minimally invasive, repeatable access to tumor-dynamics and are used for early cancer detection (although population-level screening remains challenging), disease staging and minimal residual disease monitoring, prediction of metastatic progression, real-time tracking of tumor evolution and therapeutic resistance, and assessment of treatment efficacy [5].

In parallel, mounting evidence indicates that the human microbiome, a complex ecosystem comprising bacteria, fungi, archaea and viruses, plays a major role in cancer initiation, progression, prognosis, and treatment response [6]. While the human microbiome has been linked to many chronic and immune-mediated diseases, its role in cancer remains comparatively underexplored. Although evidence suggests microbial communities may influence carcinogenesis, tumor progression, and treatment responses, systematic mechanistic studies in cancer remain limited compared to other microbiome-associated diseases, highlighting a significant gap in our understanding. The gut microbiota, in particular, can exert both pro- and anti-tumorigenic effects depending on its composition and functional activity, influencing carcinogenesis, inflammation, metabolism, and immune surveillance [7, 8]. Recent studies have underscored its importance in shaping responses to cancer immunotherapies, raising the possibility that microbiome-targeted strategies could modulate disease trajectories in metastatic setting [9–11]. Alterations in local microbiota have also emerged as potential diagnostic or prognostic markers. In pancreatic cancer, Farrel et al*.* first reported significant differences in the oral microbiome between patients and healthy controls [12]. In breast cancer, both mammary and gut microbiota appeared relevant. Distinct microbial signatures differentiated healthy breast tissue from tumor tissue, and microbial profiles may vary according to breast cancer molecular subtypes, suggesting subtype-specific microbial involvement and potential biomarkers utility [13]. Additionally, gut dysbiosis has been associated with breast cancer through its influence on estrogen metabolism and enterohepatic circulation [14]. This relationship is particularly relevant in hormone receptor-positive breast cancer given the established link between endogenous estrogen levels and postmenopausal breast cancer risk [15]. Intestinal dysbiosis is further linked to immune dysfunction, chronic inflammation, and compromised intestinal barrier integrity. Microbial products originating from the gut may reach distant organs, including the breast, via the bloodstream and contribute to tumor development [16]. However, the extent to which bacterial translocation into the systemic circulation is involved in breast cancer remains poorly understood. Thus, quantifying circulating cell-free microbial DNA (cf-mbDNA) represents an innovative approach to breast cancer management. If validated, this would broaden the concept of liquid biopsy to include analysis of the circulating microbiome in blood or “*liquid microbiopsy*”, combining cf-mbDNA with host-derived biomarkers of microbial translocation and intestinal barrier dysfunction. Such markers include soluble CD14 (sCD14) and lipopolysaccharide-binding protein (LBP), which reflect systemic immune responses to microbial components, and intestinal fatty acid-binding protein (I-FABP), a marker of enterocyte damage and intestinal permeability. These biomarkers have previously been investigated in inflammatory and autoimmune diseases [17–21], but their relevance in oncology, and particularly in breast cancer, remains largely unexplored.

The aim of this study was therefore to quantify cf-mbDNA and indirect markers of microbial translocation (sCD14, LBP, and I-FABP) in three distinct populations: healthy donors, patients with early-stage breast cancer (EBC), and metastatic breast cancer patients (MBC) previously evaluated for CTCs and PD-L1^+^-CTC quantification [22]. We aimed to determine ability of these biomarkers to *i)* enable early detection of breast cancer, *ii)* discriminate early from metastatic disease, and *iii)* predict progression and survival in MBC.

## Methods

### Patients

This retrospective study evaluated three distinct populations.

Data and samples from the 58 patients of the EBC cohort were collected at the Institute for Cancerology of Montpellier (ICM), France, following written patient consent and in accordance with French regulations. Patients were enrolled in the BCB INSTITUT clinical trial (NCT03449264) approved by the national ethics committee (Comité de Protection des Personnes Sud-Ouest et Outre-Mer III” (reference ID-RCB: 2017-A01940-53)). Blood samples were collected by the Biological Resource Center of the Montpellier Cancer Institute (CRB-ICM, BB-0033–00059) before surgical resection of the primary tumor, and before any neoadjuvant treatment.

Patients with histologically confirmed MBC, irrespective of molecular subtypes or treatment line, were enrolled in the monocentric prospective ALCINA study (AnaLysis of CIrculatiNg tumor mArkers in blood; NCT02866149), previously published [22]. Blood samples were collected before initiation of a line of MBC treatment. All participants provided written informed consent prior to study participation. Twenty healthy donors were included as controls after enrollment of cancer patients to ensure comparable age and sex distribution across groups. Plasma samples were kindly provided by Dr. Bena from the Innospecimen biobank (Clermont-Ferrand, France). All individuals had no personal history of cancer at the time of inclusion.

The study was approved by the local ethics committee (Comité de Protection des Personnes “Ile de France VI” #29–15) and conducted in accordance with Good Clinical Practice and the Declaration of Helsinki.

### Quantification of circulating cell-free microbial DNA

Circulating microbial DNA was extracted from 200 μL of plasma using the QIAamp MinElute DNA® kit (Qiagen, Courtaboeuf, France) on the QIAcube® automated extraction system (Qiagen), following the manufacturer's instructions. DNA was eluted in a final volume of 80 μl. An extraction negative control consisting of ultrapure molecular biology-grade water was processed in parallel with each batch of samples to monitor potential contamination.

Microbial DNA load was quantified in plasma by real-time qPCR targeting the 16S rRNA gene (199-bp amplicon) using a validated protocol [23]. Each sample was analyzed in duplicate, with two independent replicates. Molecular biology-grade water served as a negative control in each run. Absolute quantification relied on a standard curve generated from serial dilutions of plasmid DNA containing known copy numbers of 16S rRNA gene. Two points of the standard curve were included in each PCR run to validate amplification efficiency. The mean qPCR efficiency was 1.9, with an estimated error of 1.46%. The detection limit was 10 copies per reaction. The assay demonstrated high reproducibility across serial dilutions, with a coefficient of variation < 3.3% for all concentrations tested [24]. qPCR assays were run on the LightCycler® 480 II (Roche Diagnostics, Meylan, France), and absolute quantification analysis was carried out using Lightcycler® 480 software (Roche), version 1.5.

### Indirect markers of microbial translocation and intestinal permeability

Plasma levels of LBP and sCD14 were quantified as indirect biomarkers of microbial translocation and innate immune activation. I-FABP was quantified as a biomarker of enterocyte damage and increased intestinal permeability. LBP concentrations were determined using a commercial enzyme-linked immunosorbent assay (ELISA) kit (Enzo Life Sciences, New York, USA), according to the manufacturer's instructions. Plasma samples were diluted 1:800, and the assay detection limit was 3.125 ng/mL. sCD14 plasma levels were measured using a specific ELISA kit (Enzo Life Sciences), following the manufacturer's protocol. Samples were diluted 1:200., with a detection limit of 1.5 ng/mL. I-FABP concentrations were assessed using an ELISA kit (Bio-Techne, Minneapolis, USA), according to the manufacturer's recommendations. Plasma samples were diluted 1:2, and the detection limit was 47 pg/mL. Ultrapure water negative controls and reference serum samples were included in each assay to ensure analytical validity and reproducibility.

### C-reactive Protein measurement

Plasma C-reactive protein (CRP) concentrations were measured using a latex-enhanced immunoturbidimetric assay on a Cobas c702 automated biochemistry analyzer (Roche Diagnostics). Reagents (ref. 07876414190) and calibrators (ref. 11,355,279,216) were used according to the manufacturer’s recommendations. The analytical measurement range was 0.6–350 mg/L. Calibration was performed with each reagent batch change. Analytical imprecision remained < 5% for CRP concentrations ranging from 3 to 284 mg/L.

### CTC enumeration and PD-L1 characterization

CTC enumeration and assessment of PD-L1 expression on CTCs were performed as previously described [25]. Briefly, CTCs were isolated and enumerated using the CellSearch® system (Menarini Silicon Biosystems, Castel Maggiore, Italia), following the manufacturer’s instructions. CTCs were defined as EpCAM-positive, cytokeratin-positive, DAPI-positive, and CD45-negative cells detected in 7.5 mL of whole blood. PD-L1 expression on CTCs was evaluated using an anti-PD-L1 antibody (ref. 130–122–813; Milteniy Biotec, Bergisch Gladbach, Germany).

### Statistical analyses

Descriptive statistics are presented as medians with ranges for continuous variables and as frequencies with percentages for categorical variables. Comparisons between groups were performed using the non-parametric Wilcoxon or Kruskal–Wallis tests for continuous variables, and the Chi-square or Fisher’s exact test for categorical variables, as appropriate.

Discriminative performance of biomarkers (single or combined) was evaluated using receiver operating characteristic (ROC) curve analysis. The area under the curve (AUC) and its 95% confidence interval (95% CI) were calculated (an AUC being considered statistically significance when its 95% CI did not include 0.5). Three- and five-biomarker combinations were generated as composite biomarkers using the mROC program [26]. The selected biomarkers were combined into a single linear score (virtual marker) optimized to maximize the area under the ROC curve according to the generalized mROC criterion [27]. Optimal cut-off values were determined using Youden’s index. Sensitivity, specificity, positive and negative predictive values were subsequently calculated.

Survival analyses were restricted to the MBC cohort. Median follow-up was estimated using the reverse Kaplan–Meier method. Progression-free survival (PFS) and overall survival (OS) were estimated using the Kaplan–Meier curves and compared using the Log-rank test. PFS was defined as the time from blood sampling to the first documented disease progression or death from any cause. OS was defined as the time from blood sampling to death from any cause. The follow-up cut-off date was April 2023. Multivariate analysis was performed using Cox proportional hazards regression models (stepwise procedure). Variables with a *p*-value < 0.20 in univariate analysis were entered into the initial multivariate model. Results were expressed as hazard ratios with 95% confidence intervals (95% CIs). All statistical tests were two-sided, and *p*-values ≤ 0.05 were considered statistically significant. Statistical analyses were performed using Stata version 16.0 (StataCorp LLC, College Station, TX, USA).

## Results

### Patient characteristics

A total of 136 patients were included (Table 1), comprising 58 patients with MBC, 58 with EBC, and 20 healthy controls. The EBC and MBC cohorts were matched for age and tumor biological characteristics, including estrogen receptor (ER), progesterone receptor (PR), and HER2 (Human Epidermal growth factor Receptor-2) status.

**Table 1:** Baseline characteristics of the study population

	**Metastatic breast cancer (*N* = 58)**		**Early breast cancer (*N* = 58)**		**Healthy Controls (*N* = 20)**	
	N	%	N	%	N	%
Age (years)						
Median [min–max]	65.0	[34.8–86.8]	65.0	[42–85]	67.5	[65–70]
< 65	29	50.00	28	48.28	0	0.00
≥ 65	29	50.00	30	51.72	20	100.0
HR/HER2 status						
HR-/HER2-	8	13.79	8	13.79		
HR +/HER2-	38	65.52	38	65.52		
HER2 +	12	20.69	12	20.69		
pT						
pT1			40	68.97		
pT2			17	29.31		
pT3			1	1.72		
pN						
pN0			42	72.41		
pN1			12	20.69		
pN2			3	5.17		
pN3			1	1.72		
No. metastatic lines						
≤ 3	25	43.10				
> 3	33	56.90				
No. metastatic sites						
≤ 2	24	41.38				
> 2	34	58.62				
Total no. CTC						
median [min–max]	7	[0–4510]				
< 5	27	46.55				
≥ 5	31	53.45				
Total no. PD-L1 + CTC						
median [min–max]	0	[0–14]				
0 (negative)	43	74.14				
> 0 (positive)	15	25.86				
Biomarkers (*) (median [min–max])						
sCD14 (ng/ml)	4.75	[2.77; 8.51]	6.51	[1.51; 17.72]	5.03	[2.29; 9.45]
LBP (ng/ml)	31.85	[2.85; 52.35]	8.30	[0.59; 76.21]	10.20	[0.36; 28.68]
I-FABP (pg/ml)	310.36	[99; 2296.67]	177.0	[17; 4820.75]	160.13	[20.75; 3585.75]
16S RNA (copies)	10.56	[6.12; 81.8]	8.09	[3.75; 92.6]	6.18	[2.66; 14.44]
CRP (mg/L)	4.28	[0.34; 79.06]	1.72	[0.16; 53.44]	0.72	[0.16; 3.06]

*Abbreviations*: *HR* Hormone Receptors, *CTC* Circulating tumor cells

(*) *p-values* (non-parametric Wilcoxon test) for comparisons of biomarker distributions between groups were as follows: Metastatic *vs* Early breast cancer: *p* < 0.001 (sCD14, LBP, CRP), *p* = 0.005 (I-FABP) and *p* = 0.002 (16S RNA); Early breast cancer *vs* Healthy controls: *p* = 0.127 (sCD14), *p* = 0.810 (LBP), *p* = 0.693 (I-FABP), *p* = 0.069 (16S RNA) and *p* = 0.003 (CRP)

### Performance of circulating biomarkers for EBC detection

First, the ability of individual and combined biomarkers to discriminate patients with EBC from healthy controls was assessed. Among all biomarkers tested, CRP exhibited the highest discriminative performance. Using a Youden index-derived cut-off of 1.12 mg/L, CRP achieved an AUC of 0.73, indicating moderate discrimination between EBC patients and healthy individuals. No other single biomarker, including the Cf-mbDNA, demonstrated superior performance, and linear combinations incorporating 3-or 5-markers did not improve the AUC beyond that obtained with CRP alone. The results are summarized in Supplementary Table 1.

### Performance of circulating biomarkers for discriminating EBC and MBC

**Fig. 1:**
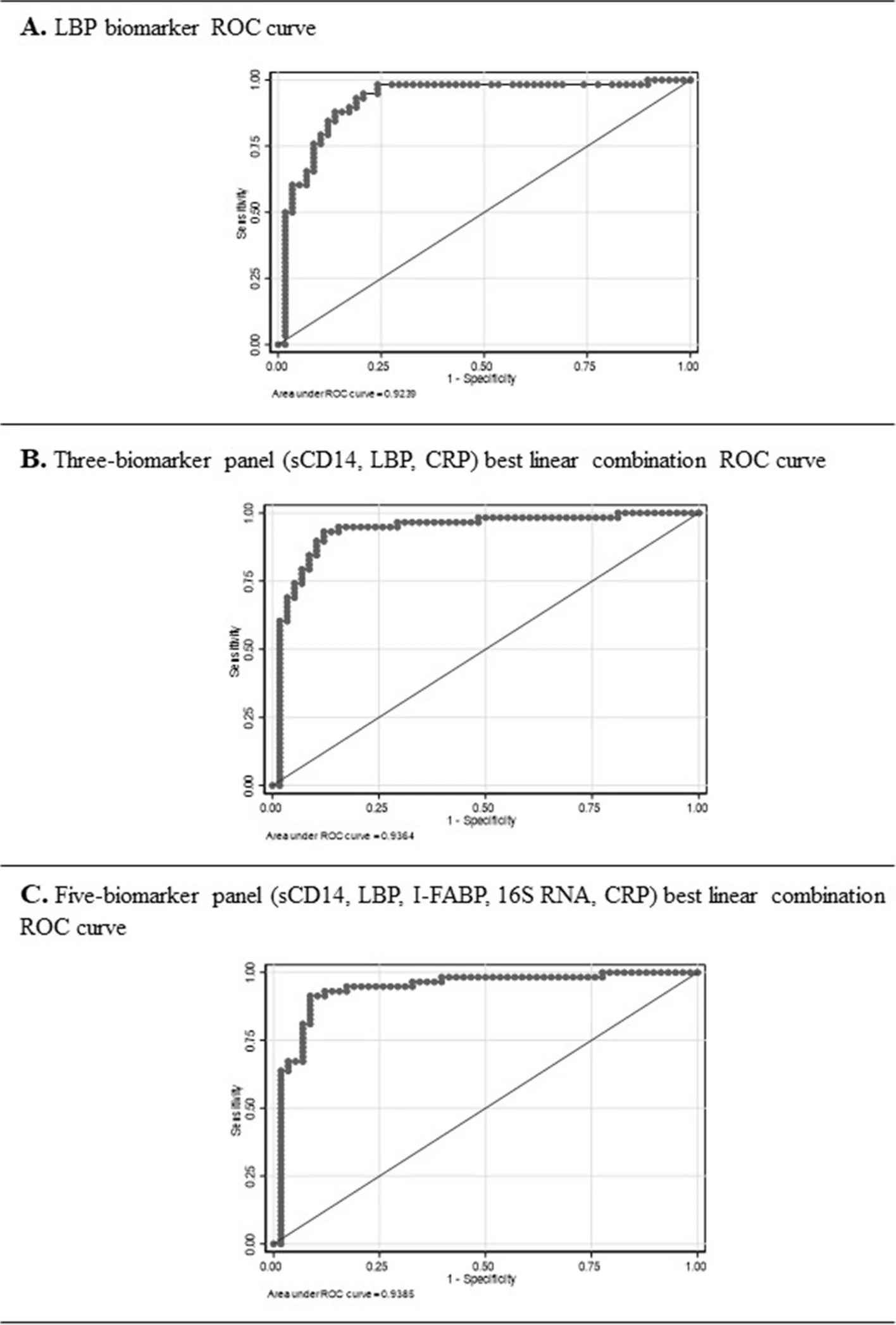
Receiver-operating characteristic (ROC) analysis for stage discrimination. ROC curves comparing metastatic breast cancer (MBC) *vs* early breast cancer (EBC) based on circulating biomarkers. The area under the curve (AUC) with 95% confidence intervals is shown for each marker; LBP is the best single classifier (AUC = 0.92). Multimarker linear models (3- and 5-marker) provide only marginal AUC improvement (0.936–0.939). *Abbreviations*: LBP, lipopolysaccharide-binding protein; sCD14, soluble cluster of differentiation 14; I-FABP, intestinal fatty acid–binding protein; CRP, C-reactive protein; cf-mbDNA, cell-free microbial DNA; AUC, area under the ROC curve

Next, the ability of the candidate biomarkers to discriminate between EBC and MBC was assessed. The corresponding results are presented in Fig. 1 and Supplementary Table 2. Among the biomarkers evaluated, LBP showed the strongest discriminative capacity, reaching an AUC of 0.92 when used as a single marker. In contrast to the EBC versus healthy control comparison, combining biomarkers provided only limited additional benefit, with 3- and 5-marker linear models yielding AUC values of 0.936 and 0.939, respectively. Although cf-mbDNA concentrations were significantly higher in MBC compared to EBC patients (*p* = 0.002) Table 1, the discriminative ability of cf-mbDNA alone at the individual level remained modest (AUC = 0.67 [0.57–0.77]), above chance but below the threshold generally considered clinically meaningful (AUC > 0.70).

### Clinicopathological correlates of biomarkers levels in MBC

To explore the clinical relevance of the candidate biomarkers in the MBC cohort, we analyzed their associations with established clinicopathological variables in this population (Supplementary Table 3). Lower CRP levels were significantly associated with HER2-positive tumors status and with fewer detected CTCs. Higher sCD14 levels were observed in patients with HR (hormone receptor)^+^/HER2^−^ tumors (*p* = 0.046). No other significant associations were identified between biomarker levels and clinicopathological variables.

### Prognostic value of circulating biomarkers in MBC-PFS

At the time of analysis, with a median follow-up of 77.5 months (95%CI: 44.9–78.0), 53/58 patients (91.4%) had experienced disease progression and 45 patients (77.6%) had died. Median PFS was 4.5 months (95%CI: 3.2–7.6).

**Fig. 2:**
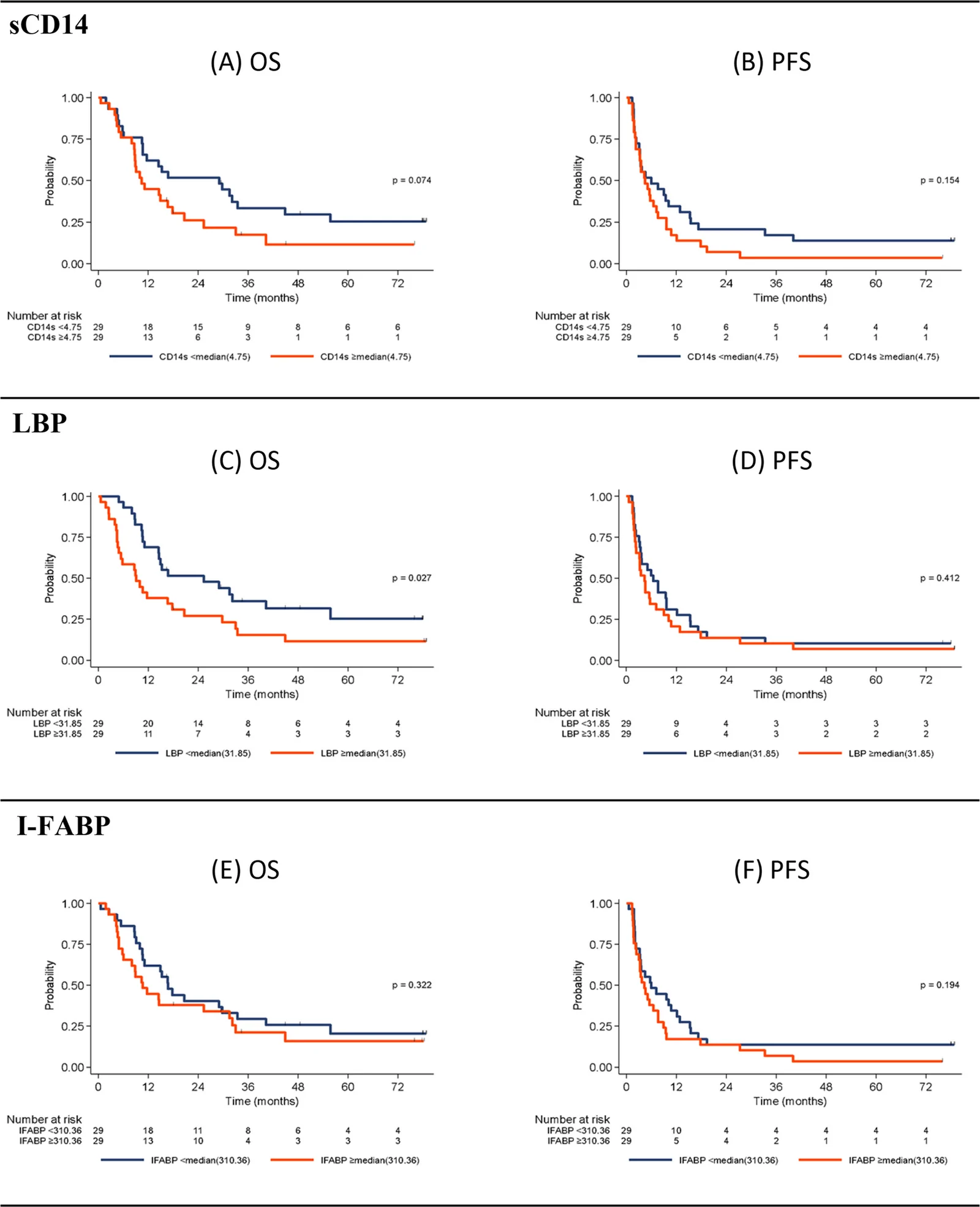

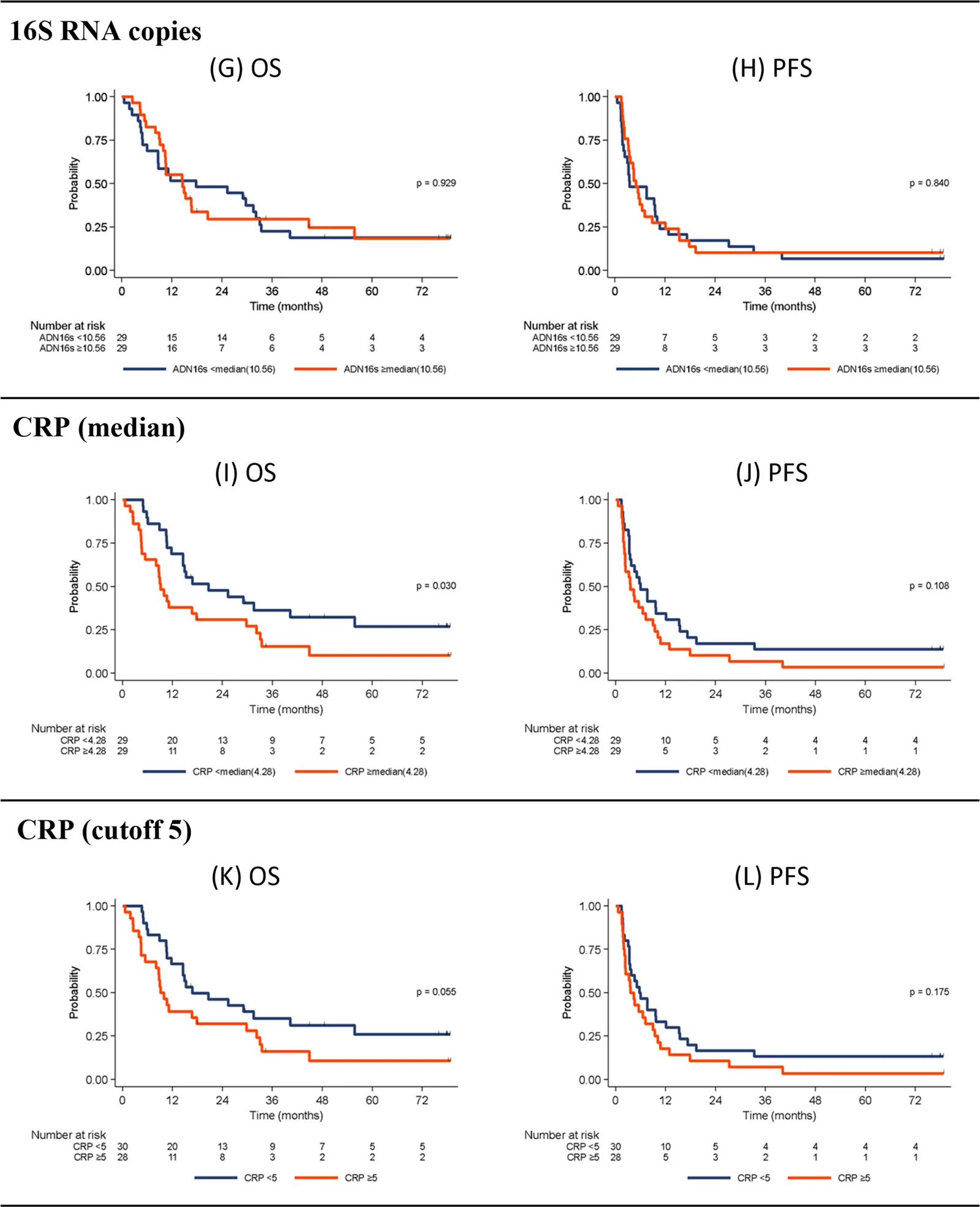
Survival analyses according to circulating biomarkers in metastatic breast cancer (*N* = 58). Kaplan–Meier overall survival (OS) and progression-free survival (PFS) curves stratified by biomarker level (median used as cutoff) (**A**) and (**B**) sCD14; (**C**) and (**D**) LBP; (**E**) and (**F**), I-FABP; (**G**) and (**H**), 16S RNA copies; (**I**) and (**J**) CRP median; (**K**) and (**L**) CRP cut-off 5 mg/L. *p*-values are from log-rank tests. In multivariate Cox models, sCD14 (OS and PFS) and I-FABP (PFS) remain independently associated with outcome

In univariate analysis (Table 2), shorter PFS was significantly associated with tumor biological subtype (*p* = 0.0005), more than three prior metastatic treatment lines (p < 0.001), presence of more than two metastatic sites (*p* = 0.01), a CTCs count ≥ 5 using the conventional threshold (*p* = 0.021), and detection of PD-L1^+^-CTCs (p < 0.001). None of the candidate circulating biomarkers was significantly associated with PFS in univariate analysis (Table 2, Fig. 2).

**Table 2:** Univariate analysis for progression-free and overall survival in metastatic breast cancer (*N* = 58)

Variables	PFS		OS	
	Hazard Ratio	95% CI	Hazard Ratio	95% CI
Age (years)				
< 65	1		1	
≥ 65	0.98	0.57–1.69	0.78	0.43–1.40
	*p* = 0.955		*p* = 0.399	
HR/HER2 status				
HR-/HER2-	1		1	
HR +/HER2-	0.56	0.25–1.23	0.53	0.24–1.17
HER2 +	0.15	0.05–0.45	0.19	0.06–0.57
	***p* = 0.0005**		***p* = 0.006**	
No. metastatic lines				
≤ 3	1		1	
> 3	4.19	2.15–8.16	3.47	1.73–6.89
	***p* < 0.001**		***p* < 0.001**	
No. metastatic sites				
≤ 2	1		1	
> 2	2.59	1.46–4.65	2.17	1.16–4.07
	***p* = 0.001**		***p* = 0.015**	
Total no. CTC				
< 5	1		1	
≥ 5	1.93	1.11–3.37	2.83	1.51–5.26
	***p* = 0.021**		***p* = 0.001**	
Total no. CTC PDL1				
0 (negative)	1		1	
> 0 (positive)	3.42	1.79–6.55	4.99	2.56–9.76
	***p* < 0.001**		***p* < 0.001**	
sCD14 (ng/ml)				
< 4.75	1		1	
≥ 4.75	1.49	0.86–2.57	1.75	0.94–3.12
	*p* = 0.157		p = 0.077	
LBP (ng/ml)				
< 31.85	1		1	
≥ 31.85	1.25	0.73–2.15	1.93	1.07–3.47
	*p* = 0.414		***p* = 0.029**	
I-FABP (pg/ml)				
< 310.36	1		1	
≥ 310.36	1.43	0.83–2.46	1.34	0.75–2.41
	*p* = 0.197		*p* = 0.324	
16S RNA (copies)				
< 10.56	1		1	
≥ 10.56	0.95	0.55–1.63	0.97	0.54–1.75
	*p* = 0.840		*p* = 0.930	
CRP (mg/L)				
< 4.28	1		1	
≥ 4.28	1.55	0.90–2.67	1.91	1.06–3.45
	*p* = 0.112		***p* = 0.033**	

Univariate Cox regression examining associations between baseline variables and progression-free survival (PFS) and overall survival (OS). Results are presented as Hazard Ratio (95% CI) with *p*-values

*Abbreviations*: *95% CI* 95% confidence interval, *CTC* Circulating tumor cells

In multivariate analysis (Table 3), tumor biological subtype, number of prior metastatic treatment lines, number of metastatic sites, detection of PD-L1^+^-CTCs, as well as circulating sCD14 and I-FABP levels, were independently associated with PFS.

**Table 3:** Multivariate analysis for progression-free survival (PFS) and overall (OS) in metastatic breast cancer population (*N* = 58)

**Variables** ^(1)^	**PFS**		**OS**	
	Hazard Ratio	95% CI	Hazard Ratio	95% CI
HR/HER2 status				
HR-/HER2-	1		1	
HR +/HER2-	0.30	0.11–0.82	0.42	0.18–0.96
HER2 +	0.14	0.04–0.50	0.24	0.08–0.76
	*p* = 0.010		*p* = 0.050	
No. metastatic lines				
≤ 3	1		1	
> 3	3.72	1.74–7.98	3.71	1.76–7.82
	*p* = 0.0003		*p* = 0.0003	
No. metastatic sites				
≤ 2	1			
> 2	3.31	1.41–7.77		
	*p* = 0.0045			
Total no. CTC				
< 5			1	
≥ 5			3.02	1.54–5.95
			*p* = 0.001	
Total no. PD-L1 + CTC				
0	1		1	
1 +	2.01	1.02–3.95	4.61	2.28–9.36
	*p* = 0.049		*p* = 0.0001	
sCD14				
< 4.75	1		1	
≥ 4.75	2.14	1.16–3.96	2.30	1.17–4.54
	*p* = 0.015		*p* = 0.017	
I-FABP				
< 310.36	1			
≥ 310.36	1.93	1.04–3.57		
	*p* = 0.036			

Cox proportional hazards models including variables with p < 0.10 in univariate analysis Results are shown as Hazard Ratio (95% CI) with *p*-values

^(1)^Variables kept in the model by a stepwise procedure; *p*-values from a Likelihood-ratio test

### Prognostic value of circulating biomarkers in MBC-OS

Median OS in the MBC cohort was 14.5 months (95%CI: 10.4–25.3). In univariate analysis (Table 2), poorer OS was significantly associated with tumor biological subtype (*p* = 0.006), more than three prior metastatic treatment lines (p < 0.001), more than two metastatic sites (*p* = 0.015), a CTC count ≥ 5 (*p* = 0.001), and detection of PD-L1^+^-CTCs (p < 0.001). In addition, higher LBP (*p* = 0.029) and higher CRP levels, using a threshold of 4.28 mg/L (*p* = 0.038), were significantly associated with shorter OS (Fig. 2).

In multivariate analysis (Table 3), tumor biological subtype, number of prior metastatic treatment lines, CTCs count, detection of PD-L1^+^-CTCs, and sCD14 levels remained independently associated with OS.

## Discussion

**Fig. 3:**
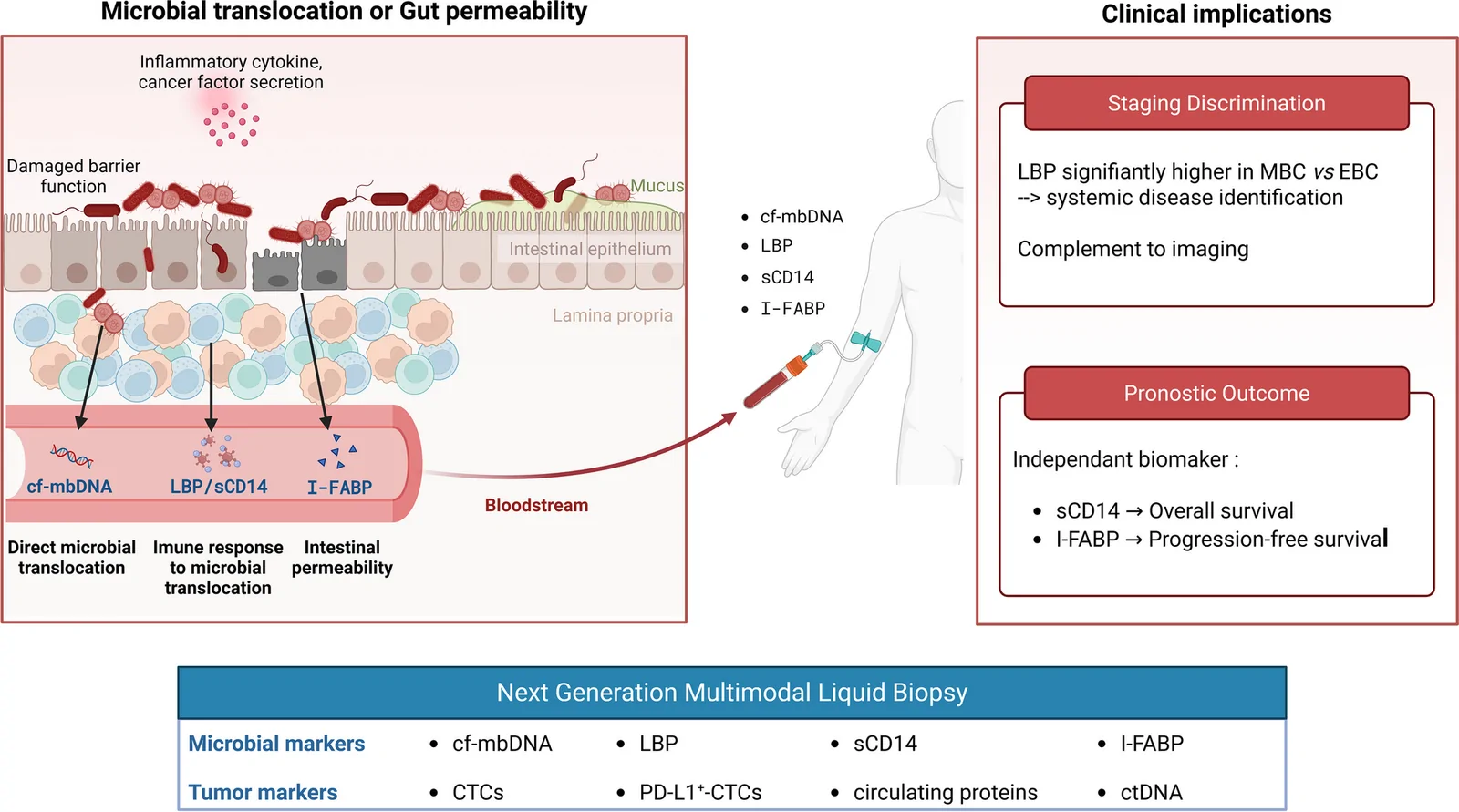
The liquid microbiopsy concept and its clinical implications in breast cancer. Gut barrier dysfunction leads to microbial translocation, resulting in the presence of circulating microbial DNA (cf-mbDNA) and host response markers (LBP, sCD14, I-FABP). These biomarkers form a “liquid microbiopsy” that complements conventional liquid biopsy approaches. Translocation markers discriminate early from metastatic disease, while host response markers provide independent prognostic information in metastatic breast cancer. However, their limited performance for early detection highlights the need for multimodal strategies integrating microbial, host, and tumor-derived analytes

In this study, levels of circulating biomarkers associated with microbial translocation, most notably LBP, demonstrated marked differences between EBC and MBC, while host-response markers (sCD14, I-FABP) were independently associated with outcomes in MBC. In contrast, performance for detecting EBC versus healthy donors was modest, consistent with the known limits of non-tumor-specific inflammatory markers in screening settings. Together, these findings support the concept of a “*liquid microbiopsy*” [28], in which cf-mbDNA and translocation/permeability markers complement conventional liquid biopsy analytes (CTCs/ctDNA) to refine staging and prognostication, in line with the current multimodal evolution of liquid biopsy in oncology [29, 30] Fig. 3.

A growing body of evidence links the gut and mammary microbiota to breast cancer carcinogenesis, immune modulation, and treatment response [31, 32]. Distinct microbial signatures have been reported between healthy and malignant breast tissues, with variations across molecular subtypes [33–35]. Gut dysbiosis may influence disease through the estrobolome, which modulates enterohepatic circulation of estrogens, an especially relevant mechanism in hormone receptor–positive tumors, although human data remain heterogeneous and mechanism-level evidence continues to evolve [32, 36, 37].

Our findings are biologically coherent with a model in which increased intestinal permeability facilitates bacterial-product translocation, leading to innate immune activation and systemic inflammation that may promote more aggressive tumor behavior. LBP and sCD14 are established indicators of microbial-ligands exposure (e.g., lipopolysaccharide) and monocyte activation, while I-FABP reflects enterocyte damage. These markers are widely used as surrogates of barrier dysfunction in inflammatory and immune-mediated conditions [17–20, 23, 38]. Notably, the correlation between LBP and the lactulose/mannitol permeability test further supports LBP as a practical surrogate of gut barrier integrity in clinical studies [39]. CRP was included alongside these translocation-specific markers as a reference indicator of systemic inflammation, given its widespread clinical use. Although CRP is not specific to microbial translocation, it reflects inflammatory pathways that are closely interconnected with, and frequently activated downstream of, host responses to microbial components. Combining CRP with LBP, sCD14, and 16S rDNA therefore allowed us to assess whether integrating markers reflecting distinct but biologically related aspects of systemic inflammation and host–microbial interaction could improve discrimination between patient groups.

Beyond local tissue effects, circulating microbial signatures have been associated with cancer biology and therapeutic outcomes in other contexts. In melanoma, cf-mbDNA profiles and sCD14 concentrations have been linked to immunotherapy response, underscoring the translational potential of microbiome-derived signals [40]. Our study extends these observations by suggesting that host-response markers (sCD14, I-FABP) provide independent prognostic information in MBC, complementing tumor-intrinsic measures such as CTC counts and PD-L1^+^- CTCs [22, 41–43].

We confirmed the prognostic relevance of CTCs (≥ 5/7.5 mL) and the added information provided by PD-L1^+^-CTCs, consistent with findings across solid tumors and growing interest in their use as dynamic immunotherapy [30, 42, 43]. At the same time, studies report distinct mammary and gut microbiome patterns in breast cancer with subtype-specific differences, although significant heterogeneity in sampling and analytical methods underscores the need for standardized protocols. Our observation of higher sCD14 in HR^+^/HER2^−^ tumors is consistent with a hormone-immune-microbiome crosstalk hypothesis, which warrants testing in controlled human and preclinical models [33, 34, 44]. It would also be of interest to assess whether the prognostic value of sCD14 and I-FABP differs across molecular subtypes in MBC; however, the limited number of patients within each subgroup (*n* = 8 TNBC, *n* = 12 HER2^+^) precludes statistically meaningful subgroup analyses in the current dataset. This question warrants investigation in larger, molecularly stratified cohorts.

Regarding cf-mbDNA and the “sterile blood” paradigm, large population-based analyses in healthy individuals have found no evidence for a stable core blood microbiome, instead supporting a model of sporadic, transient, low-amplitude microbial translocation from other body sites [45]. Consistent with this, cf-mbDNA concentrations were significantly higher in MBC compared to EBC patients (*p* = 0.002); however, the discriminative ability of cf-mbDNA alone remained modest (AUC = 0.67 [0.57–0.77]), indicating that while a group-level difference exists, cf-mbDNA quantification alone is insufficient for reliable individual-level classification. This may reflect: *i)* genuinely low concentrations of circulating microbial DNA across disease stages; *ii)* the cross-sectional design, which may miss transient translocation events; and *iii)* pre-analytical and low-biomass constraints, including handling time, freeze–thaw conditions, and decontamination variability, which can obscure small between-group differences. In contrast, host-response markers (LBP, sCD14, I-FABP) appeared to provide more consistent signals than cf-mbDNA quantification alone, suggesting that host inflammatory pathways may capture biologically relevant information related to disease staging and prognosis. However, these findings remain preliminary due to the limited sample size and require validation in larger, adequately powered cohorts before definitive conclusions can be drawn regarding their clinical utility. This further highlights the added value of integrating host response markers, which provided superior discriminative performance compared to cf-mbDNA alone in our cohort. As LBP and sCD14 are both partly liver-derived, we examined whether the presence of liver metastases, reported in 34 of the 58 MBC patients (58.6%), influenced their circulating levels; no significant difference was observed for either marker between patients with and without liver metastases (LBP: *p* = 0.949; sCD14: *p* = 0.239), suggesting that hepatic involvement did not substantially confound our findings. Although underexplored in breast cancer, LBP has been associated with outcomes in hepatocellular and gastric cancers and with radiation-related late effects, supporting its relevance as a systemic endotoxemia marker with potential prognostic value [46, 47].

Potential clinical implications emerge along three axes: *i)* Staging discrimination: LBP, alone or with additional translocation markers, strongly differentiates EBC from MBC and may help identify patients with a higher likelihood of systemic disease biology when imaging findings are inconclusive [30]; *ii)* Prognosis in MBC: sCD14 (and I-FABP for PFS) provides independent prognostic value, complementing tumor-centric markers (CTCs, PD-L1 on CTCs) and potentially informing treatment intensity or supportive care [22, 41]; *iii)* Screening/early detection: no translocation markers alone or in linear combinations provided screening-appropriate performance versus healthy donors in our dataset. Multimodal liquid biopsy integrating cf-mbDNA, proteins, ctDNA, and potentially tumor-derived proteomics, may be required to reach acceptable sensitivity/specificity thresholds [29].

Strengths of our work include the quality and size of the biobanks, which enabled a high degree of matching with detailed clinical and pathological patient data, as well as a multimodal translational design integrating simultaneous assessment of cf-mbDNA, LBP, sCD14, I-FABP, CRP, CTCs, and PD-L1 on CTCs. Limitations include the retrospective design of the MBC cohort, limited sample size and tumor biology heterogeneity for individualized prediction, the unavailability of BMI data for the MBC cohort and healthy controls precluding a comprehensive assessment of its potential confounding effect on LBP levels (which were significantly higher in obese EBC patients, *p* = 0.007, while sCD14 did not differ significantly, *p* = 0.365), the taxonomic and viability limits of pan-bacterial qPCR, and the intrinsic susceptibility of cf-mbDNA assays to low-biomass artefacts despite rigourous controls. This limitation was moderated by following low-biomass recommendations, namely using multiple negative controls (extraction/PCR blanks), batch randomization, reagent-lot tracking, spike-ins, and transparent reporting of filtering workflows [48–50]. Furthermore, the qPCR approach targeting a 200-bp fragment of the 16S rRNA gene enables sensitive quantification of cf-mbDNA but does not allow taxonomic characterization of the bacterial genera contributing to the detected signal. Short-read shotgun metagenomic sequencing of plasma samples could be implemented in future studies to provide a comprehensive analysis of circulating bacterial DNA and offer deeper insights into its origin and diversity across disease stages. Nevertheless, due to these limitations, these results must be considered as exploratory, and independent analyses using larger cohorts are mandatory to strengthen these data.

## Conclusions

Beyond conventional tumor-derived liquid-biopsy signals (CTCs, ctDNA), our results show that a measurable host–microbiota–barrier axis (LBP, sCD14, I-FABP, cf-mbDNA) provides additional stage-related and prognostic information in breast cancer, particularly in the metastatic setting. Rather than supporting the existence of a stable blood microbiome, these findings are consistent with transient microbial translocation that may exacerbate systemic inflammation and contribute to poorer outcomes. Within this paradigm, liquid microbiopsy, combining cf-mbDNA with host-responses markers, appears as a complementary extension of liquid-biopsy deserving validation with rigorous low-biomass contamination control and standardized pre-analytical workflows.

Looking ahead, integrating liquid microbiopsy with conventional liquid biopsy approaches could redefine how we monitor tumor–host interactions and refine risk stratification in breast cancer. Larger, multicenter studies with standardized, contamination-controlled workflows will be essential to validate its clinical robustness and determine whether these signals can guide therapeutic decisions. Ultimately, capturing both tumor-derived and host–microbiota dynamics may offer a more holistic and actionable view of disease progression and patient outcome.

## Supplementary Information

Supplementary Material 1. Supplementary Table 1. ROCbased univariate and multivariate analyses for early breast cancer (EBC) vs healthy controls. Singlemarker and multimarker model performance with AUC (95% CI), optimal Youden cutoffs, sensitivity, specificity, and predictive values. Supplementary Table 2. ROCbased univariate and multivariate analyses for metastatic breast cancer (MBC) vs early breast cancer (EBC). Singlemarker and multimarker model performance with AUC (95% CI); LBP shows the highest singlemarker discrimination. Supplementary Table 3. Correlations between circulating biomarkers and baseline clinicopathological variables in metastatic breast cancer (MBC). Associations tested by nonparametric methods as appropriate; significant findings include lower CRP in HER2+ tumors and higher sCD14 in HR+/HER2− tumors.

## Data Availability

The datasets used and/or analysed during the current study are available from the corresponding author on reasonable request.

## Abbreviations

ALCINA: AnaLysis of CIrculatiNg tumor mArkers
AUC: Area under the curve
cf-mbDNA: Cell-free microbial DNA
CRP: C-reactive protein
CTC: Circulating tumor cell
ctDNA: Cell-free tumor DNA
DAPI: 4’,6-Diamidino-2-phenylindole
EBC: Early-stage breast cancer
ELISA: Enzyme-linked immunosorbent assay
EpCAM: Epithelial cell adhesion molecule
ER: Estrogen receptor
HER2: Human Epidermal growth factor Receptor-2
HR: Hormone receptor
I-FABP: Intestinal fatty acid-binding protein
LBP: Lipopolysaccharide-binding protein
MBC: Metastatic breast cancer patients
PD-L1: Programmed death-ligand 1
PFS: Progression-free survival
PR: Progesterone receptor
OS: Overall survival
qPCR: Quantitative Polymerase Chain Reaction
ROC: Receiver operating characteristic
sCD14: Soluble CD14
CI: Confidence interval
