# Supplementary material for: Circulating blood microbiome biomarkers as liquid biopsy in breast cancer
Source: J Exp Clin Cancer Res. 2026 Aug 11;45:230. doi: 10.1186/s13046-026-03782-8 (PMC13741746; doi:10.1186/s13046-026-03782-8)
Supplement: Supplementary file 1 [file 13046_2026_3782_MOESM1_ESM.docx]

**Supplementary Table 1.**

| **Early breast cancer (EBC) vs Control** | | | | | | |
| --- | --- | --- | --- | --- | --- | --- |
| ROC Analysis | Optimal threshold | AUC [95% CI] | Se | Sp | PPV | NPV |
| sCD14 | 5.89 | 0.62 [0.48 ; 0.75] | 0.59 | 0.70 | 0.85 | 0.37 |
| LBP | 8.62 | 0.52 [0.37 ; 0.70] | 0.53 | 0.60 | 0.79 | 0.31 |
| I-FABP | 297 | 0.53 [0.39 ; 0.67] | 0.33 | 0.85 | 0.86 | 0.30 |
| 16S RNA | 4.86 | 0.64 [0.48 ; 0.79] | 0.91 | 0.40 | 0.82 | 0.62 |
| CRP | 1.12 | 0.73 [0.60 ; 0.85] | 0.66 | 0.70 | 0.86 | 0.41 |
|  |  |  |  |  |  |  |
| **Biomarker best-lineal combination:** | | | | |  |  |
| **3-biomarker panel:** | | | | |  |  |
| +0.0588 × (sCD14)  + 0.019 × (16S RNA)  + 0.0372 × (CRP) | 0.659 | 0.694 [0.57 ; 0.82] | 0.48 | 0.85 | 0.90 | 0.36 |
|  |  |  |  |  |  |  |
| **5-biomarker panel:** | | | | |  |  |
| +0.0822 × (sCD14)  - 0.0327 × (LBP)  + 0.000138 × (I-FABP) + 0.0196 × (16S RNA)  + 0.0752 × (CRP) | 0.549 | 0.732 [0.62 ; 0.85] | 0.66 | 0.80 | 0.90 | 0.44 |

*Optimal threshold maximizing Youden index. AUC: Area under the ROC curve; 95% CI: 95% confidence interval of the AUC estimation (an AUC is considered statistically significance when its 95% CI does not include 0.5); Se: Sensitivity; Sp: Specificity; PPV: Positive predictive value; NPV: Negative predictive value

**Supplementary Table 2.**

| **Metastatic breast cancer (MBC) versus Early breast cancer (EBC)** | | | | | | |
| --- | --- | --- | --- | --- | --- | --- |
| ROC Analysis | Optimal threshold | AUC [95% CI] | Se | Sp | PPV | NPV |
| sCD14 | 6.30 | 0.69 [0.59 ; 0.79] | 0.81 | 0.52 | 0.63 | 0.73 |
| LBP | 14.0 | 0.92 [0.87 ; 0.98] | 0.95 | 0.76 | 0.80 | 0.94 |
| I-FABP | 232.5 | 0.65 [0.55 ; 0.75] | 0.64 | 0.64 | 0.64 | 0.64 |
| 16S RNA | 8.78 | 0.67 [0.57 ; 0.77] | 0.71 | 0.59 | 0.63 | 0.67 |
| CRP | 3.58 | 0.73 [0.64 ; 0.82] | 0.59 | 0.81 | 0.76 | 0.66 |
|  |  |  |  |  |  |  |
| **Biomarker best lineal combination:** | | | | |  |  |
| **3- biomarker panel:** | | | | |  |  |
| -0.211 × (sCD14)  + 0.108 × (LBP)  - 0.0389 × (CRP) | 0.487 | 0.936 [0.89 ; 0.99] | 0.91 | 0.88 | 0.88 | 0.91 |
|  |  |  |  |  |  |  |
| **5- biomarker panel:** | | | | |  |  |
| -0.219 × (sCD14)  +0.108 × (LBP)  - 09.56e-05 × (I-FABP)  + 0.006 × (16S RNA)  - 0.0393 × (CRP) | 0.663 | 0.939 [0.89 ; 0.99] | 0.90 | 0.91 | 0.91 | 0.90 |

*Optimal threshold maximizing Youden index. AUC: Area under the ROC curve; 95% CI: 95% confidence interval of the AUC estimation (an AUC is considered statistically significance when its 95% CI does not include 0.5); Se: Sensitivity; Sp: Specificity; PPV: Positive predictive value; NPV: Negative predictive value

**Supplementary Table 3.**

(Med= median value of the biomarker)

| Variables  (MBC cohort) |  | **sCD14** | | **LBP** | | **IF-ABP** | | **16S RNA** | | **CRP** | | |
| --- | --- | --- | --- | --- | --- | --- | --- | --- | --- | --- | --- | --- |
|  | N | Med^(1)^ | *p* | Med^(1)^ | *p* | Med^(1)^ | *p* | Med^(1)^ | *p* | Med^(1)^ | *p* |  |
| Age (years) |  |  | 0.680 |  | 0.150 |  | 0.174 |  | 0.059 |  | 0.076 |  |
| <65 | 29 | 4.36 |  | 35.06 |  | 283.22 |  | 9.97 |  | 8.63 |  |  |
| ≥65 | 29 | 4.76 |  | 28.19 |  | 380.83 |  | 12.34 |  | 3.58 |  |  |
| ER status |  |  | 0.366 |  | 0.124 |  | 0.356 |  | 0.595 |  | 0.263 |  |
| - (<10%) | 13 | 4.21 |  | 20.71 |  | 492.5 |  | 10.14 |  | 3.8 |  |  |
| + (≥10%) | 45 | 4.81 |  | 33.36 |  | 305.83 |  | 10.76 |  | 6.44 |  |  |
| PR status |  |  | 0.591 |  | 0.692 |  | 0.709 |  | 0.388 |  | 0.508 |  |
| - (<10%) | 28 | 4.50 |  | 33.21 |  | 361.25 |  | 11.22 |  | 5.41 |  |  |
| + (≥10%) | 30 | 4.79 |  | 31.60 |  | 294.53 |  | 10.16 |  | 4.07 |  |  |
| HER2 status |  |  | 0.185 |  | 0.057 |  | 0.803 |  | 0.565 |  | **0.017** |  |
| - | 46 | 4.90 |  | 34.22 |  | 318.33 |  | 10.56 |  | 6.62 |  |  |
| + | 12 | 4.08 |  | 23.51 |  | 287.92 |  | 10.84 |  | 1.82 |  |  |
| Biological subtype |  |  | **0.046** |  | 0.057 |  | 0.611 |  | 0.738 |  | **0.034** |  |
| Triple Negative | 8 | 3.80 |  | 24.70 |  | 496.67 |  | 8.55 |  | 4.03 |  |  |
| HR+/HER2- | 38 | 5.04 |  | 34.94 |  | 307.86 |  | 10.92 |  | 6.78 |  |  |
| HER2+ | 12 | 4.08 |  | 23.51 |  | 287.92 |  | 10.84 |  | 1.82 |  |  |
| Triple Negative |  |  | 0.075 |  | 0.300 |  | 0.321 |  | 0.528 |  | 0.596 |  |
| No | 50 | 4.82 |  | 31.85 |  | 307.08 |  | 10.92 |  | 4.88 |  |  |
| Yes | 8 | 3.80 |  | 24.70 |  | 496.67 |  | 8.55 |  | 4.03 |  |  |
| No. metastatic lines |  |  | 0.363 |  | 0.604 |  | 0.535 |  | 0.525 |  | 0.296 |  |
| ≤3 | 25 | 4.97 |  | 31.55 |  | 319.17 |  | 11.53 |  | 3.67 |  |  |
| >3 | 33 | 4.56 |  | 32.16 |  | 309.89 |  | 10.01 |  | 6.66 |  |  |
| No. metastatic sites |  |  | 0.554 |  | 0.625 |  | 0.190 |  | 0.613 |  | 0.177 |  |
| ≤2 | 24 | 4.24 |  | 28.03 |  | 394.25 |  | 10.05 |  | 3.51 |  |  |
| >2 | 34 | 4.89 |  | 31.90 |  | 294.53 |  | 10.92 |  | 6.55 |  |  |
| Total no. CTC |  |  | 0.387 |  | 0.129 |  | 0.809 |  | 0.121 |  | **0.018** |  |
| <5 | 27 | 4.27 |  | 24.00 |  | 305.83 |  | 9.97 |  | 3.12 |  |  |
| ≥5 | 31 | 5.03 |  | 34.26 |  | 319.17 |  | 11.53 |  | 6.89 |  |  |
| Presence of PDL1+ CTC |  |  | 0.224 |  | 0.743 |  | 0.965 |  | 0.838 |  | 0.211 |  |
| No | 43 | 4.76 |  | 28.55 |  | 305.83 |  | 11.09 |  | 3.84 |  |  |
| Yes | 15 | 4.00 |  | 35.06 |  | 337.5 |  | 10.01 |  | 7.24 |  |  |

^(1)^ Med: Median value of the biomarker in each subgroup (defined by each clinical variable)
